# Immunomodulatory role of Parkinson’s disease 7 in inflammatory bowel disease

**DOI:** 10.1038/s41598-021-93671-1

**Published:** 2021-07-16

**Authors:** Rita Lippai, Apor Veres-Székely, Erna Sziksz, Yoichiro Iwakura, Domonkos Pap, Réka Rokonay, Beáta Szebeni, Gábor Lotz, Nóra J. Béres, Áron Cseh, Attila J. Szabó, Ádám Vannay

**Affiliations:** 1 1st Department of Pediatrics, Semmelweis University, 54, Bókay Street, Budapest, 1083 Hungary; 2 ELKH-SE Pediatrics and Nephrology Research Group, Budapest, Hungary; 3 Research Institute for Biomedical Sciences and Center for Animal Disease Models, Research Institute for Science and Technology, Tokyo University of Science, Tokyo, Japan; 4 2nd Department of Pathology, Semmelweis University, Budapest, Hungary

**Keywords:** Molecular biology, Inflammatory bowel disease, Crohn's disease, Ulcerative colitis, Molecular medicine

## Abstract

Recently the role of Parkinson’s disease 7 (PARK7) was studied in gastrointestinal diseases, however, the complex role of PARK7 in the intestinal inflammation is still not completely clear. Expression and localization of PARK7 were determined in the colon biopsies of children with inflammatory bowel disease (IBD), in the colon of dextran sodium sulphate (DSS) treated mice and in HT-29 colonic epithelial cells treated with interleukin (IL)-17, hydrogen peroxide (H_2_O_2_), tumor necrosis factor (TNF)-α, transforming growth factor (TGF)-β or lipopolysaccharide (LPS). Effect of PARK7 on the synthesis of IBD related cytokines was determined using *PARK7* gene silenced HT-29 cells and 3,4,5-trimethoxy-N-(4-(8-methylimidazo(1,2-a)pyridine-2-yl)phenyl)benzamide (Comp23)—compound increasing PARK7 activity—treated mice with DSS-colitis. PARK7 expression was higher in the mucosa of children with Crohn’s disease compared to that of controls. While H_2_O_2_ and IL-17 treatment increased, LPS, TNF-α or TGF-β treatment decreased the PARK7 synthesis of HT-29 cells. *PARK7* gene silencing influenced the synthesis of *IL1B, IL6*, *TNFA* and *TGFB1* in vitro. Comp23 treatment attenuated the ex vivo permeability of colonic sacs, the clinical symptoms, and mucosal expression of *Tgfb1*, *Il1b*, *Il6* and *Il10* of DSS-treated mice. Our study revealed the role of PARK7 in the regulation of IBD-related inflammation in vitro and in vivo, suggesting its importance as a future therapeutic target*.*

## Introduction

IBD, including Crohn’s disease (CD) and ulcerative colitis (UC) is a chronic inflammatory disorder of the gastrointestinal tract that dramatically impacts the quality of life. IBD starting in childhood leads to a life-long disease, which is frequently accompanied by serious complications^1–3^. The incidence and prevalence of IBD are rapidly increasing and has become a major public health problem worldwide. Although the pathogenesis of IBD has not been fully elucidated the role of oxidative stress is indisputable. Indeed, chronic inflammation is accompanied by the continuous generation of reactive oxygen species (ROS), damaging the integrity of mucosal epithelial layer. Enhanced mucosal permeability promotes the invasion of immunogenic elements, including bacteria, which in turn further facilitate the local inflammation and ROS generation.

PARK7 is a small, ubiquitously expressed homodimer protein that was primarily studied regarding the central nervous system. Indeed, mutations of PARK7 have been proved to be associated with autosomal recessive early-onset Parkinson’s disease^4^, and its protective role was suggested in Alzheimer’s disease^5^ and stroke^6^, as well. Protective effect of PARK7 is related at least in part to its antioxidant effects. PARK7 quenches the effect of ROS through the oxidation of its cysteine residues, however more importantly it increases the expression of mitochondrial uncoupling proteins^7^, thus reducing the production of mitochondrial ROS. PARK7 also induces the synthesis of antioxidant enzymes superoxide dismutase 1 and 3, thus facilitating degradation of ROS^8,9^. Moreover, PARK7 was reported to stabilize nuclear factor erythroid-2-related factor 2 (Nrf2), the master transcription factor of oxidative stress^10^. However, accumulating studies reveal that besides its antioxidant effects, PARK7 has multiple functions: it has chaperone activity, it can inhibit the abnormal protein aggregation characteristic for neurodegenerative disorders and it has also been reported to be involved in ubiquitin–proteasome system^6^. In addition, recently anti-inflammatory effect of PARK7 was demonstrated via the inhibition of antigen-induced TNF-α and IL-4 production of mast cells^11^.

The above considerations, and also our previous studies demonstrating the role of PARK7 in the maintenance of small intestinal mucosal integrity^12,13^ led us to study the role of PARK7 in the pathogenesis of IBD. In this study we report the presence, regulation and role of PARK7 in the pathomechanism of mucosal inflammation using tissue samples of therapy-naive children with IBD, and in vitro and in vivo experimental models of colitis. Our study suggests the possible therapeutic relevance of PARK7 in the treatment of IBD.

## Materials and methods

### Patients

Children with newly diagnosed IBD (CD: n = 27 (15 for PCR, 19 for WB), UC: n = 18 (10 for PCR, 7 for WB)) and controls (n = 19 (10 for PCR, 10 for WB)) were enrolled in the present study (Table 1). IBD was diagnosed according to the “Porto criteria”^14,15^, and activity score was evaluated according to the Pediatric Crohn’s Disease or Ulcerative Colitis Activity Index (PCDAI or PUCAI)^16,17^. Colonic biopsy samples were taken from macroscopically inflamed (iCD) and non-inflamed (iCD) colonic mucosa regions of children with CD, and from inflamed colonic mucosa regions of children with UC. Controls were referred with chronic abdominal pain, diarrhoea or polyposis and their colonic biopsy showed normal macroscopic and histological appearance.

**Table 1:** Clinical characteristics of the study population in the Control, Crohn’s Disease (CD) and Ulcerative Colitis (UC) groups.

	Control	CD	UC
Number (for PCR, for WB)	19 (10, 10)	27 (15, 19)	18 (10, 7)
Age (years), median (range)	6.24 (1–16)	13.59 (2–18)^#^	11.61 (1–18)^#^
Gender (boys/girls)	7/12	11/16	8/10
BMI z-score, median (range)	− 0.25 (− 3.47–2.67)	− 2.04 (− 5.79–0.61)^#^	− 0.39 (− 2.84–1.36)
Activity score, median (range)^a^	–	23.8 (5–50)^#^	38.06 (15–60)^#^
Iron (umol/l), median (range)	15.76 (8–32)	4.73 (1–20)^#^	8.11 (1–22)^#^
Albumin (g/l), median (range)	46.556 (37–68)	37.72 (26–46)^#^	40.94 (18–48)^#^
Trombocyta (Giga/l), median (range)	369.21 (210–530)	499.38 (151–837)^#^	461.83 (158–656)
CRP (mg/l), median (range)	0.96 (0–5)	31.23 (1–134.6)^#^	5.76 (0–31)^#^

*BMI* body mass index, *CRP* C-reactive protein.

^a^Activity score: CD/UC Activity Index.

^#^p < 0.05 *vs*. control.

### DSS-induced mouse model of colitis and Comp23 treatment

Experiments were performed on 7–8 weeks old, male C57Bl/6 J (WT; Charles River Laboratories, Sulzfeld, Germany) and *Il17*−/− mice^18^. Animals were housed in a temperature-controlled (22 ± 1 °C) room with alternating light and dark cycles and had free access to standard rat chow and water.

To investigate the effect of IL-17 on the synthesis of PARK7 wild type (WT) and *Il17*−/− mice were randomized into control groups receiving drinking water (0 day; n = 6/group) or DSS-treated groups gained drinking water containing 2.5% DSS (w/v; MP Biomedicals, LLC, Santa Ana, CA, USA) until the termination of the experiment on 3rd, 5th or 7th days (3, 5 or 7 days; n = 6/group). Colon samples were collected under general anesthesia induced by inhalation of isoflurane mixed with air using a vaporizer (Eickemeyer Veterinary Equipment Ltd., Twickenham, UK).

To investigate the effect of PARK7 on the pathomechanism of DSS-induced colitis, WT animals were treated with Comp23 (Enamine, Riga, Latvia), a PARK7-binding compound, that increase its activity by preventing the excessive oxidation of 106 cysteine residue of PARK7 protein, for 10 days^19^.

In this set of experiments mice were randomized into four groups receiving solvent (DMSO diluted with 20% physiology saline, i.p.; n = 6; Control group), Comp23 (10 mg/ttkg/die Comp23 dissolved in solvent, i.p.; n = 6; Comp23 group), DSS (as described above; n = 6; DSS group) or DSS and Comp23 (as described above; n = 6; DSS + Comp23 group). After the 7th day DSS was replaced by water in the DSS and DSS + Comp23 groups. Disease activity index (DAI) of animals were determined until the 10th day of the treatments. For molecular biological analyses the colon samples of another set of mice were collected on the 7th day of the experiment (Table 2).

**Table 2:**
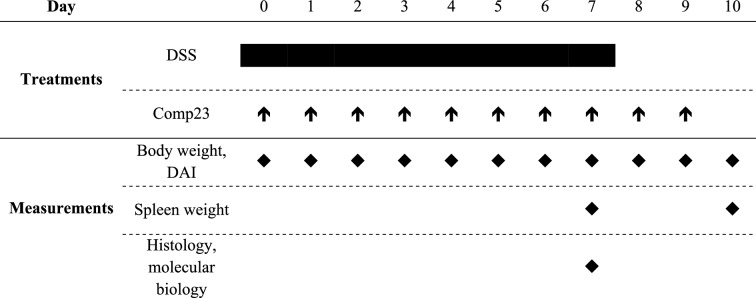
Experimental setup investigating the effect of Comp23 in DSS-induced colitis of WT mice.

During the experiment, WT mice received water for 10 days (Control and Comp23 groups) or 2.5% DSS in their drinking water for 7 days, then clear water for 3 days (DSS and DSS + Comp23 groups). Mice were treated with daily by intraperitoneal injection of Comp23 (Comp23 and DSS + Comp23 groups) or vehicle only (Control and DSS groups). Changes in the body weight of mice and disease activity index (DAI) were monitored during the whole experiment. Experiments were termined on the 7th or 10th day. Spleen weights were measured from samples derived from 7 and 10th days. Molecular biological measurements were performed on tissue samples derived from 7th day.

### Determination of weight loss and DAI

Changes in the body weight of mice were monitored during the whole experiment and measured on a digital scale in the same hour every day. DAI was determined as described in the literature previously^20^. Briefly, clinical signs of colitis including stool consistency and stool blood were scored from 0 to 3, and general state of animals was determined on a scale from 0 to 2 for 10 days. Total scores were calculated as the sum of values of the three categories. Body-weight loss and spleen weight of animals were also determined.

### Ex vivo mucosal permeability measurement

Intestinal permeability was measured based on Mateer et al.^21^. Briefly, 2 cm long intestinal sacs were prepared from the colon of DMSO (Control) and Comp23 pretreated (10 mg/kg, intraperitoneally, 1 h before experiment) WT mice (n = 3–4/group). The intestinal sacs were equally filled with FITC-conjugated 4 kDa dextran (0.1 mg/ml; SigmaAldrich) and Rhodamine B-conjugated 70 kDa dextran (0.1 mg/ml; Sigma-Aldrich) diluted either in Dulbecco’s modified Eagle medium (DMEM; Thermo Fisher Scientific, Waltham, MA, USA) or in DMEM supplemented with H_2_O_2_ (1000 μM) and placed into 5 ml phosphate-buffered saline (PBS) at 37 °C. The permeation of dextrans was determined in every 20 min for 4 h. At the end of the experiment, the sacs were opened to determine the remnant dextran release (100%). Fluorescence intensities of PBS samples were measured at λ_exc_: 485 nm, λ_em_﻿: 535 nm (Fluorescein) or λ_exc_: 544 nm, λ_em_: 632 nm (Rhodamine B) in a Hidex Chameleon Microplate Reader (Triathler, Plate Chameleon, 300SL Lablogic Systems, Inc., Brandon, FL, USA) using the MikroWin 2000 program.

### Immunocytochemistry and immunofluorescence staining

HT-29 cells were seeded in chambers and cultured for 24 h at 37 °C. After repeated washing, cells were permeabilized with Cytofix/Cytoperm (BD Pharmingen, San Diego, CA, USA) for 15 min at room temperature (RT), and were washed again. Human frozen colon biopsy samples were embedded into Shandon cryomatrix (ThermoElectron Co., Madison, WI, USA) and cut into 5 μm sections. Thereafter cells or tissue sections were incubated with rabbit anti-human PARK7 or IL-17R polyclonal IgG primary antibody (Abcam, Cambridge, United Kingdom) (1:200) for 1 h at RT. Cells were washed with WashPerm solution, and tissue sections with PBS, thereafter incubated with Alexa Fluor 488 or 568 labelled chicken anti-rabbit IgG secondary antibody (1:200, Invitrogen, Life Technologies, Carlsbad, CA, USA) for 30 min at RT in the dark and then counterstained with Hoechst 33342 (1:2000, Sigma-Aldrich, USA) for 10 min. Finally, slides were coverslipped with Vectashield fluorescent mounting medium (Vector Laboratories, Burlingame, CA, USA). Appropriate controls were performed by omitting the primary antibodies to assure their specificity and to avoid autofluorescence. Fluorescence signals were analysed with a Nikon C2 confocal laser scanning microscope system (Nikon, Minato, Tokyo, Japan).

### Histology

Paraffin sections of paraformaldehyde (4%, pH 7.4) fixed colon samples of mice were stained with hematoxylin–eosin or periodic acid–Schiff methods. Images were taken with Olympus IX81 microscope system (Olympus Corporation, Tokyo, Japan). Briefly, histological signs of colitis including (1) cell-rich subepithelial regenerative foci (granulation tissue), (2) mucosal infiltration of neutrophils, (3) oedema under the epithelial layer, (4) erosion and (5) vacuolation of epithelial cells were determined on a scale from 0 to 6, blindly by an expert pathologist. Histology score values were then calculated as the sum of scores of the above mentioned five categories.

### HT-29 colonic epithelial cells and treatments

HT-29 human colonic epithelial cell line (Sigma-Aldrich) was cultured in modified McCoy’s 5A medium (Sigma-Aldrich) supplemented with l-glutamine, 10% heat-inactivated fetal bovine serum (FBS) (Invitrogen), 100 U/ml penicillin and 100 µg/ml streptomycin (Sigma-Aldrich) at 37 °C and 5% CO_2_.

Cells were starved in 0% FBS for 24 h then trypsinized and seeded in 6-well plates at a density of 5 × 10^5^ cells/well, resulting in nearly 80% confluency. After plating, cells (n = 6/groups) were treated with 100 ng/ml recombinant human IL-17A (R&D Systems, Minneapolis, MN, USA), 20 µM H_2_O_2_ (Reanal, Budapest, Hungary), 10 ng/ml TNF-α (R&D Systems), 1 nM TGF-β (Life Technologies), or 100 ng/ml lipopolysaccharides (LPS) from *Escherichia coli* (Sigma-Aldrich) for 24 h at 37 °C. IL-17A, H_2_O_2_, TNF-α and LPS were dissolved in sterile PBS, TGF-ß was dissolved in 4 mM HCl. Control cells were treated only with vehicle.

To identify the IL-17-activated ROS-producing enzyme, cells were pretreated for 30 min with NAD(P)H-oxidase inhibitor, diphenyleneiodonium chlorid (DPI; 5 μM, Sigma-Aldrich) dissolved in DMSO.

### PARK7 gene silencing and IL-17 treatment of HT-29 colonic epithelial cells

Exponentially growing cells were seeded in 6-well plates (5 × 10^5^ cells/well, n = 6/group) and cultured for 24 h at 37 °C. Medium was replaced with Opti-MEM I reduced-serum medium (92.5 µl/ml, Invitrogen) containing 0% FBS, and *PARK7* specific small interfering (si)RNA (30 nM, Invitrogen) and Lipofectamine RNAiMax Transfection Reagent (2.5 µl/ml, Invitrogen). Sense sequence of PARK7 siRNA was GGUUUUGGAAGUAAAGUUAtt. As negative control, cells were treated with the same medium containing Lipofectamine and a nonsense, short scrambled (sc)RNA (30 nM, Invitrogen), which is not complementary with any gene in the target organism. Cells were incubated in transfection reagent for 24 h. After transfection, cells were treated with vehicle or with recombinant human IL-17A (100 ng/ml) for 24 h.

### RNA isolation and real-time RT-PCR

Total RNA was isolated from biopsy samples, mouse colon tissues or HT-29 colonic epithelial cells using Geneaid Total RNA Mini Kit (Geneaid Biotech Ltd., New Taipei City, Taiwan). RNA (1 μg/sample) was reverse-transcribed using Maxima First Strand cDNA Synthesis Kit for RT-qPCR (Life Technologies) to generate first-stranded cDNA. The mRNA expressions were determined by real-time RT-PCR using a Light Cycler 480 system (Roche Diagnostics, Mannheim, Germany). Nucleotide sequences, annealing temperatures of the primer pairs and resulted PCR product lengths are shown in Table 3.

**Table 3:** Nucleotide sequences of primer pairs applied for the real-time PCRs, the product length and the temperature of anellation.

Gene	Primer pairs	Product length (bp)	T_a_ (°C)
h*PARK7*	Forward: 5′-AGT ACA GTG TAG CCG TGA-3′ Reverse: 5′-TAA TCT GGG CGC ACA GAA TT-3′	116	60
h*TNFA*	Forward: 5′-GAG GCG CTC CCC AAG AAG ACA-3′ Reverse: 5′-TGG GCC AGA GGG CTG ATT AGA-3′	182	60
h*TGFB1*	Forward: 5′-GCG TGC GG CAG CTG TAC ATT GAC T-3′ Reverse: 5′-CGA GGC GCC CGG GTT ATG C-3′	174	60
h*IL1B*	Forward: 5′-CAC GCT CCG GGA CTC ACA G -3′ Reverse: 5′-GCC CAA GGC CAC AGG TAT TTT-3′	160	56
h*IL6*	Forward: 5′-AAA GAT GGC TGA AAA AGA TGG AT-3′ Reverse: 5′-CTC TGG CTT GTT CCT CAC TAC TCT-3′	146	60
h*IL10*	Forward: 5′-ATG CCC CAA GCT GAG AAC CAA GAC-3′ Reverse: 5′-AGA AAT CGA TGA CAG CGC CGT AGC-3′	107	60
h*GAPDH*	Forward: 5′-CAC CAC CAT GGA GAA GGC TG-3′ Reverse: 5′-GTG ATG GCATGG ACT GTG-3′	240	60
h*RPLP0*	Forward: 5′-GAG GCT GCC AAC CGG AAC AAT GAC-3′ Reverse: 5′-TCC TGC AGG CGG CCA ATA GTG TCT-3′	206	60
m*Park7*	Forward: 5′-TTT ATC TGA GTC GCC TAT GGT G-3′ Reverse: 5′-TTT GGA TGC AAG GTC ACA AC-3′	132	60
m*Tnfa*	Forward: 5′-GGG CCA CCA CGC TCT TCT GTC TA-3′ Reverse: 5′-GAG AGG GAG GCC ATT TGG GAA CTT-3′	83	56
m*Tgfb1*	Forward: 5′-GTG CGG CAG CTG TAC ATT GAC TTT-3′ Reverse: 5′-GGC TTG CGA CCC ACG TAG TAG AC-3′	239	59
m*Il17*	Forward: 5′-AGG ACT TCC TCC AGA ATG T-3′ Reverse: 5′-CCG CAA TGA AGA CCC TGA-3′	136	60
m*Il1b*	Forward: 5′-GCC ACC TTT TGA CAG TGA TGA GAA-3′ Reverse: 5′-GAT GTG CTG CTG CGA GAT TTG A-3′	36	55
m*Il6*	Forward: 5′-AAC CAC GGC CTT CCC TAC TTC A-3′ Reverse: 5′-TGC CAT TGC ACA ACT CTT TTC TCA-3′	155	55
m*Il10*	Forward: 5′-CAA AGG ACC AGC TGG ACA ACA TAC-3′ Reverse: 5′-GCC TGG GGC ATC ACT TCT ACC-3′	124	54
m*Gapdh*	Forward: 5′-ATC TGA CGT GCC GCC TGG AGA AAC-3′ Reverse: 5′-CCC GGC ATC GAA GGT GGA AGA GT-3′	164	60
m*Rn18s*	Forward: 5′-AGC GGT CGG CGT CCC CCA ACT TCT-3′ Reverse: 5′-GCG CGT GCA GCC CCG GAC ATC TA-3′	107	60

*h* human, *m* mouse, *bp* base pair, *Ta* annealing temperature.

The expected lenght of the PCR products were monitored by gele electrophoresis (2% agarose gel, Bioline, London, UK). The melting curves and the relative mRNA expressions were analysed by a Light-Cycler 480 software, version 1.5.0.39 (Roche) and determined by comparison with *GAPDH, RPLP0* or *18S* as internal control using the ΔΔCt method. Data were normalized and presented as the ratio of their control values.

### Protein isolation and western blotting

Colonic tissue samples from patients, mice or HT-29 colonic epithelial cells were homogenized in lysing solution, containing 50 mM HEPES, 150 mM NaCl, 1% Triton X-100, 5 mM EDTA, 5 mM EGTA, 20 mM sodium pyrophosphate, 20 mM NaF, 0.2 mg/ml phenylmethylsulfonyl fluoride, 0.01 mg/ml leupeptin, and 0.01 mg/ml aprotinin (pH 7.4) (each substance obtained from Sigma-Aldrich). Thereafter protein concentration was determined by a detergent-compatible protein assay (BioRad, Hercules, CA, USA). Denatured samples (20 μg protein/lane) were separated on 4–20% gradient SDS polyacrylamide gel (BioRad) and transferred to nitrocellulose membranes (BioRad). Pre-stained protein standard (BioRad) was used as molecular weight marker. Blot membranes were then cut in two pieces to determine the amount of PARK7 and that of the corresponding loading control from identical samples. Membranes were blocked in 1% non-fat dry milk solution for 1 h and incubated with PARK7-specific rabbit polyclonal antibody (1:300) overnight (Abcam). Equal protein loading was confirmed by β-actin specific rabbit polyclonal IgG antibody (1:2000, Santa Cruz Biotechnology Inc., Santa Cruz, CA, USA), GAPDH specific rabbit polyclonal IgG antibody (1:2000, Santa Cruz Biotechnology Inc.), or total protein amount. Then blots were incubated with horse radish peroxidase (HRP)-conjugated goat anti-rabbit IgG secondary antibody (1:2000, Santa Cruz Biotechnology Inc.) for 30 min. Immunoreactive bands were developed using the enhanced chemiluminescence Western blotting detection protocol (GE Healthcare, Little Chalfont, United Kingdom) and the resulted images were visualized with VersaDoc 5000MP system (Bio-Rad) and analyzed using Quantity One v4.6.9 (Bio-Rad) and ImageJ 1.48v (National Institutes of Health, Bethesda, MD, USA) softwares, the results were expressed as relative optical density.

### Enzyme-linked immunosorbent assay (ELISA)

Protein level of TNF-α in the lysate of PARK7 gene silenced HT-29 colonic epithelial cells was measured using human TNF-α DuoSet ELISA (R&D Systems) according to the manufacturer’s protocol. Absorbance was recorded at 450 nm and at 570 nm as background using a Hidex Chameleon Microplate Reader (Triathler, Plate Chameleion, 300SL Lablogic Systems, Inc., Brandon, FL, USA) and MikroWin 2000 software.

### Flow cytometry

HT-29 cells were centrifuged, washed with PBS and incubated for 10 min at RT with FACS Permeabilizing Solution 2 (BD Pharmingen). Permeabilized cells were washed with PBS and incubated with rabbit anti-human PARK7 polyclonal IgG primary antibody (1:25, AbCam) for 30 min at RT. Cells were subsequently washed with Permeabilizing Solution 2 and incubated with Alexa Fluor 488 chicken anti-rabbit IgG secondary antibody (1:50, Invitrogen) for 30 min at RT in dark. Negative controls were incubated with the secondary antibody alone. Thereafter cells were washed with Permeabilizing Solution 2, centrifuged and resuspended in PBS. Analysis was performed with BD FACSAria cytometer (BD Pharmingen). We identified an intact cell gate, according to the forward and side scatter. 10^4^ cells were collected and results were analysed using BD FACSDiva Software (BD Pharmingen).

### Statistical analysis

Data were analyzed using GraphPad Prism 5.0. software (GraphPad Software Inc., La Jolla, CA, USA). After testing the normality with Kolmogorov–Smirnov test, ordinary one- or two-way ANOVA- and unpaired- or multiple t-test, Mann–Whitney or Kruskall–Wallis-test were used to determine the differences among groups. Results were illustrated as mean + SEM with dots, representing individual values. P values less than 0.05 were considered to statistically significant.

### Ethical considerations

Written informed consent was obtained from parents of each participant prior to the procedure. Study was approved by the Semmelweis University Regional and Institutional Committee of Science and Research Ethics (TUKEB 58/2013) and performed in compliance with the Declaration of Helsinki.

All animal procedures were approved by the Committee on the Care and Use of Laboratory Animals of the Council on Animal Care at Semmelweis University, Budapest, Hungary (PEI/001/82-4/2013). All methods were carried out in accordance with the relevant regulations and ARRIVE guidelines.

## Results

### Presence of PARK7 in the colonic mucosa of children

**Figure 1:**
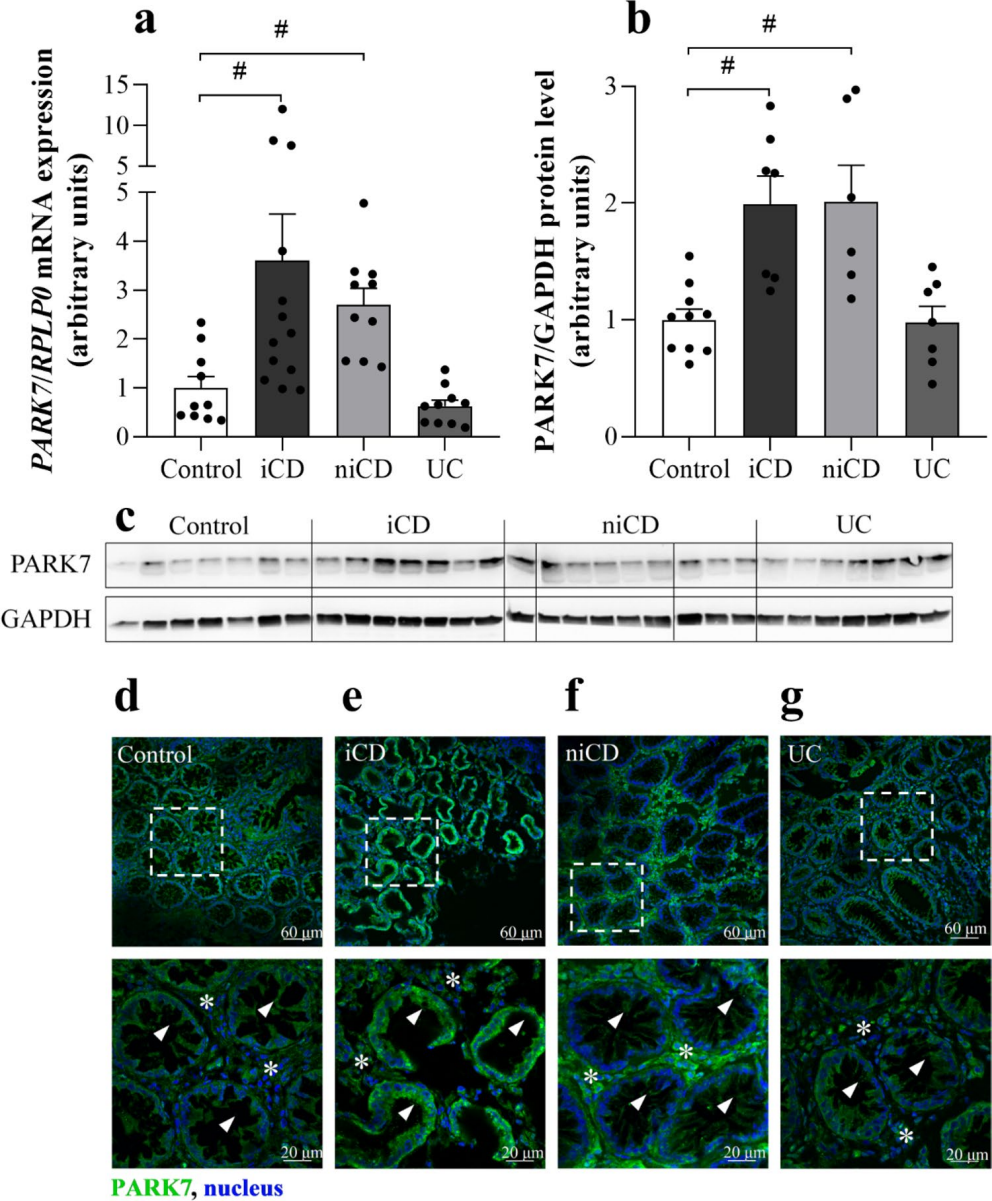
Presence of PARK7 in the colonic mucosa of children. The mRNA expression (**a**), protein level (**b**, **g**) and localization (**c**–**f**) of PARK7 were determined by real time PCR (**a**) (n = 10–13), Western blot (**b**) (n = 6–10) or immunohistochemistry (**c**–**f**, green) in the inflamed (iCD) and non-inflamed (niCD) colonic mucosa of children with Crohn’s disease (**a**, **b**, **d**, **e**), ulcerative colitis (UC) (**a**, **b**, **f**, **g**) and controls (**a**–**c**, **g**). Relative expression levels are presented in comparison with *RPLP0* mRNA (**a**) or GAPDH protein (**b**, **g**) as internal control. The data are normalized against corresponding controls**.** Results are illustrated as mean + SEM, dots represent individual values. Analysis of significance was performed by ordinary one-way ANOVA test. ^#^p < 0.05 comparing the connected groups. Full-length blots are included in “Supplementary Informations”. Cell nuclei were counterstained with Hoechst 33342 (blue). Scale bar: 60 µm or 20 µm. Arrowheads indicate crypt epithelial cells, * symbols indicate lamina propria.

The mRNA expression (Fig. 1a) and protein amount (Fig. 1b,c) of PARK7 were increased in the macroscopically inflamed (iCD) and non-inflamed (niCD) colonic mucosa of children with CD compared to controls. Amount of PARK7 remained unchanged in the colonic mucosa of UC patients. Strong PARK7 immunopositivity was observed in epithelial and lamina propria cells of children with CD (Fig. 1d–g).

### Effect of IL-17, H2O2, TNF-α, TGF-β, LPS, and DPI treatment on PARK7 synthesis of HT-29 cells

**Figure 2:**
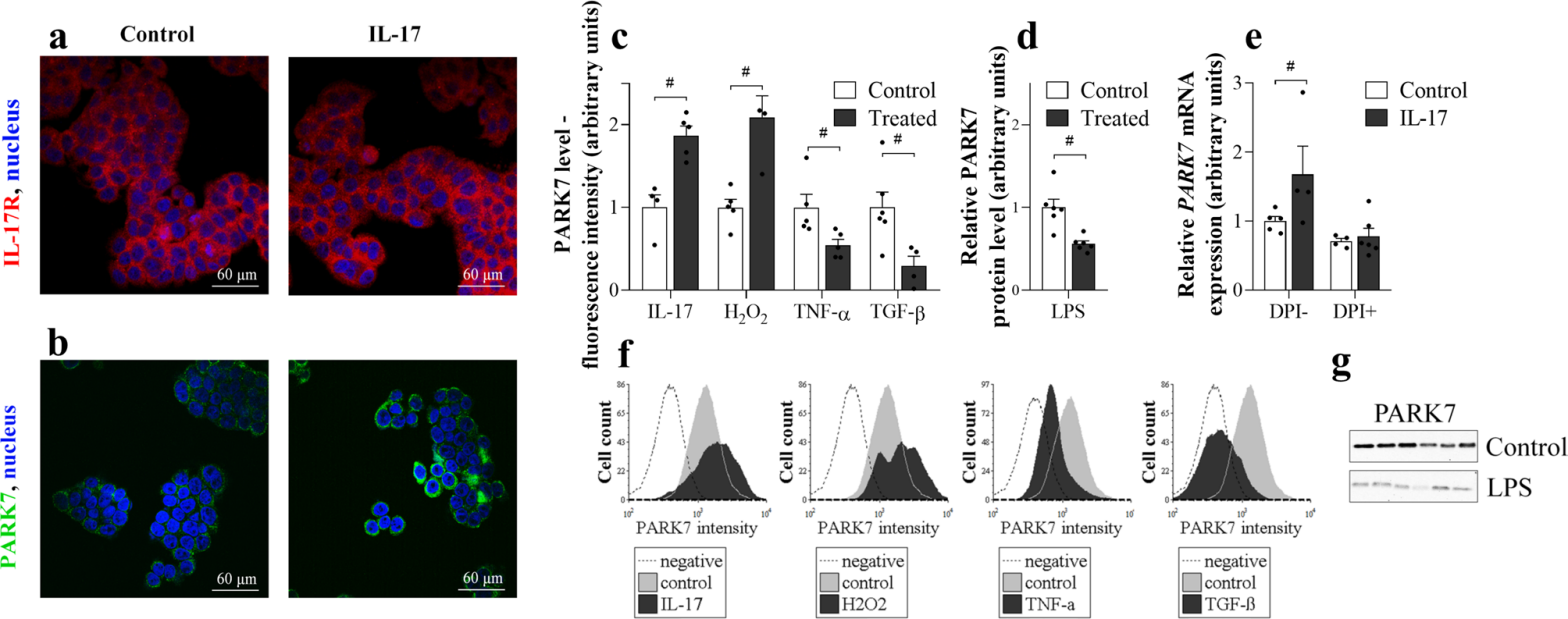
Effect of IL-17, H_2_O_2_, TNF-α, TGF-β, LPS, or DPI treatment on PARK7 synthesis of HT-29. IL-17R (**a**, red) and PARK7 (**b**, green) were visualized by immunofluorescent staining. Cell nuclei were counterstained with Hoechst 33342 (blue). Scale bar: 60 µm. PARK7 amount after various treatments (n = 4–6) was determined by flow cytometry (**c**, **f**), western blot (**d**, **g**) or real-time PCR (**e**). Relative expression levels are presented in comparison with total protein amount (**a**) or *GAPDH* mRNA (**b**) as internal control and normalized to vehicle treated group. Results are illustrated as mean + SEM, dots represent individual values. Analysis of significance was performed by Mann–Whitney test. ^#^p < 0.05 comparing the connected groups. Full-length blots are included in “Supplementary Informations”.

HT-29 cells showed IL-17R immunopositivity (Fig. 2a). While IL-17 or H_2_O_2_ treatment increased, TNF-α, TGF-β or LPS treatment decreased the amount of PARK7 (Fig. 2c,d,f,g). The increased *PARK7* expression of IL-17 treated cells was inhibited by pretreatment with DPI, a NAD(P)H oxidase inhibitor (Fig. 2e).

### Effect of DSS treatment on the colonic *Il17*, *Tnfα* and PARK7 synthesis of WT and *Il17*−/− mice

**Figure 3:**
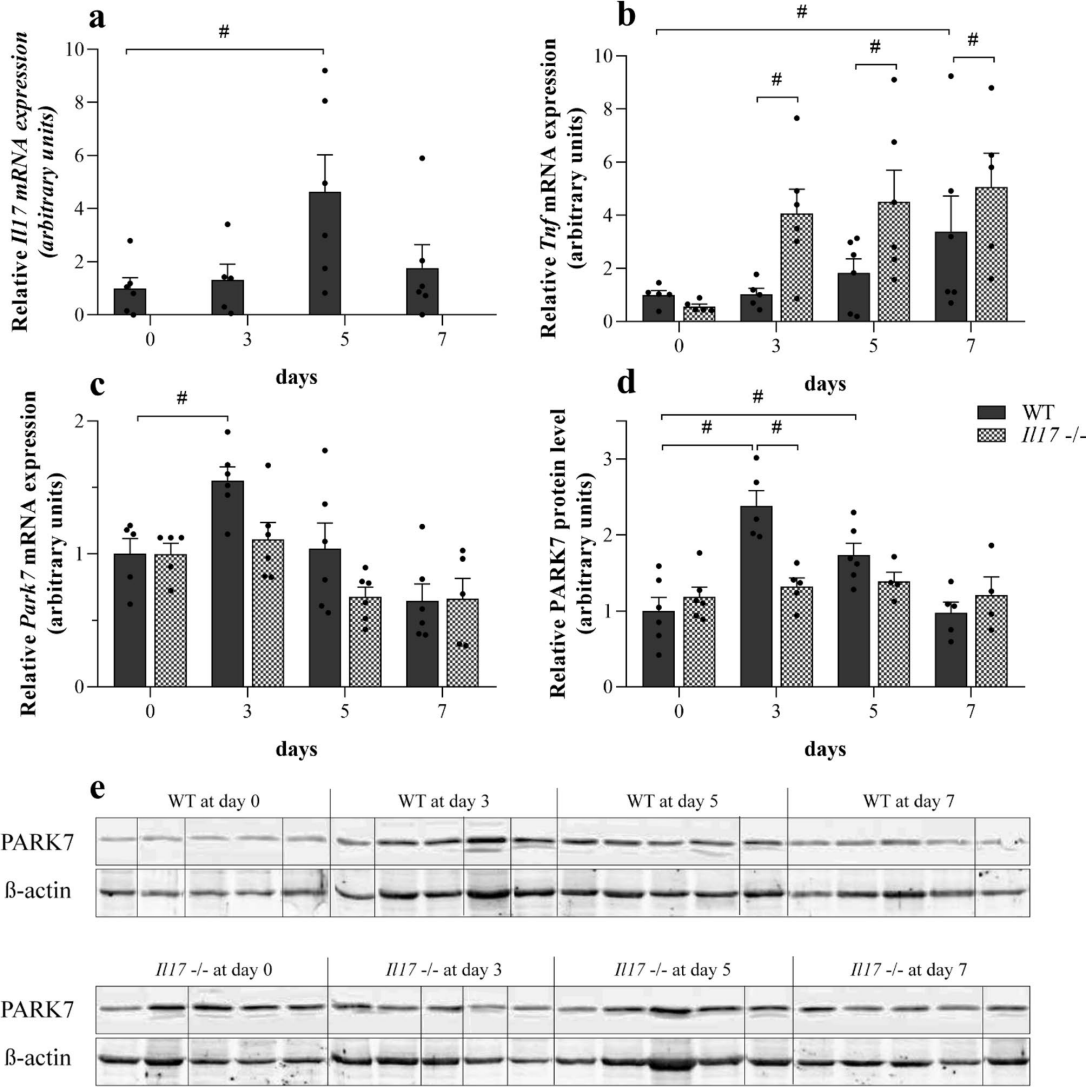
Effect of DSS on colonic *Il17*, *Tnfα* and PARK7 synthesis of WT and *Il17*−/− mice. Colonic mRNA expression of *Il17* (**a**), *Tnfa* (**b**) and *Park7* (**c**) and the protein level of PARK7 (**d**, **e**) were determined by real-time PCR (**a**–**c**) and Western blot (**d**, **e**) (n = 5–6). Relative expression levels are presented in comparison with *Gapdh* mRNA (**a**–**c**) or ß-actin protein (**d**, **e**) as internal control and normalized to WT at day 0 group. Results are illustrated as mean + SEM, dots represent individual values. Analysis of significance was performed by two-way ANOVA. ^#^p < 0.05 comparing the connected groups. Full-length blots are included in “Supplementary Informations”.

mRNA expression of *Il17* was increased on the 5th day in the colon of DSS-treated WT mice (Fig. 3a). While mRNA expression of *Tnfα* was increased only on the 7th day in the colon of WT mice, it was increased on the 3rd day and remained elevated until the end of experiment in the colon of DSS-treated *Il17*−/− mice compared to the corresponding controls (Fig. 3b).

While mRNA expression of *Park7* was increased on the 3rd day and protein level on the 3rd and 5th day in the colon of DSS-treated WT mice, it remained unchanged in the *Il17*−/− mice (Fig. 3c–e).

### Effect of PARK7 gene silencing on *TNFA*, *IL1B*, *IL6*, *IL10* and *TGFB1* expression

**Figure 4:**
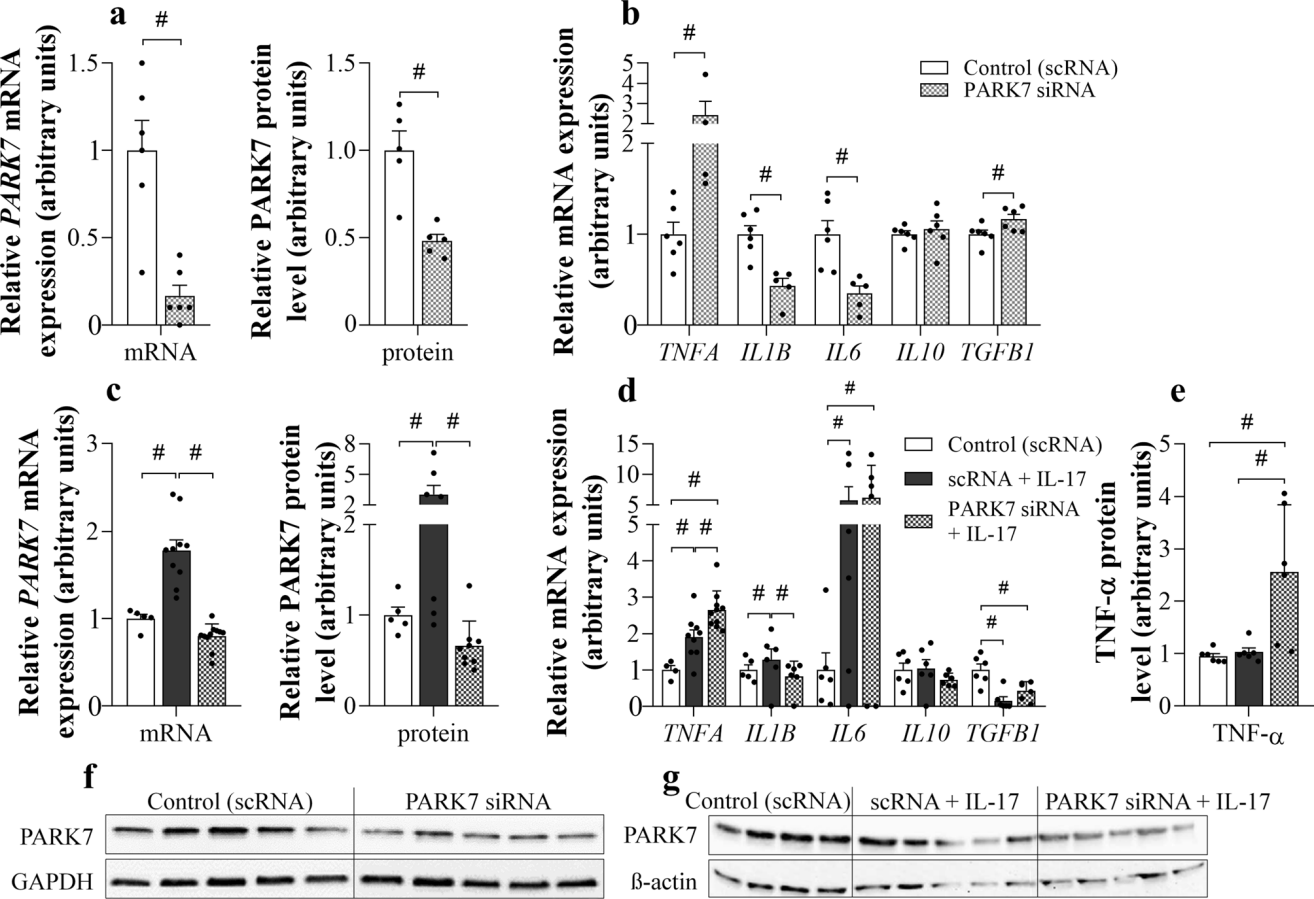
Effect of *PARK7* silencing on *TNFA*, *IL1B, IL6, IL10* and *TGFB1* expression of HT-29 cells. Cells were transfected either with scRNA or PARK7 specific siRNA (**a**–**g**) and a group of transfected cells was also treated with IL-17 (**c**–**e**, **g**). The mRNA expression of *PARK7, TNFΑ, IL1Β, IL6, IL10* and *TGFB1* was determined by real-time PCR (**a**–**d**), and the protein level of PARK7 (**a**, **c**, **f**, **g**) and TNF-α (**e**) by Western blot (**a**, **c**, **f**, **g**) or ELISA (**e**), respectively (n = 4–6). Relative expression levels are presented in comparison with *Gapdh* mRNA (**a**–**d**), GAPDH (**a**, **f**) or ß-actin protein (**c**, **g**) as internal control and normalized to vehicle treated group. Results are illustrated as mean + SEM, dots represent individual values. Analysis of significance was performed by Mann–Whitney or Kruskal–Wallis test. ^#^p < 0.05 comparing the connected groups. Full-length blots are included in “Supplementary Informations”.

Treatment with PARK7-specific siRNA resulted in decreased mRNA expression and protein level of PARK7 both in untreated and IL-17 treated HT-29 cells (Fig. 4a,c,f,g).

*PARK7* gene silencing increased mRNA expression of *TNFA*, *TGFB1* and decreased that of *IL1B* and *IL6* as compared to control cells (Fig. 4b).

IL-17 treatment increased mRNA expression and protein level of PARK7, and mRNA expression of *TNFA, IL1B, IL6*; however, decreased mRNA expression of *TGFB1* of HT-29 cells as compared to controls (Fig. 4d).

*PARK7* gene silencing further increased mRNA expression and protein level of TNF-α of IL-17 treated HT-29 cells as compared to control or IL-17 treated cells (Fig. 4d,e). Neither IL-17 treatment, nor *PARK7* gene silencing had any effect on the mRNA expression of *IL10* in colonic epithelial cells (Fig. 4b,d).

### Effect of PARK7-binding Comp23 on DSS induced colitis of mice

**Figure 5:**
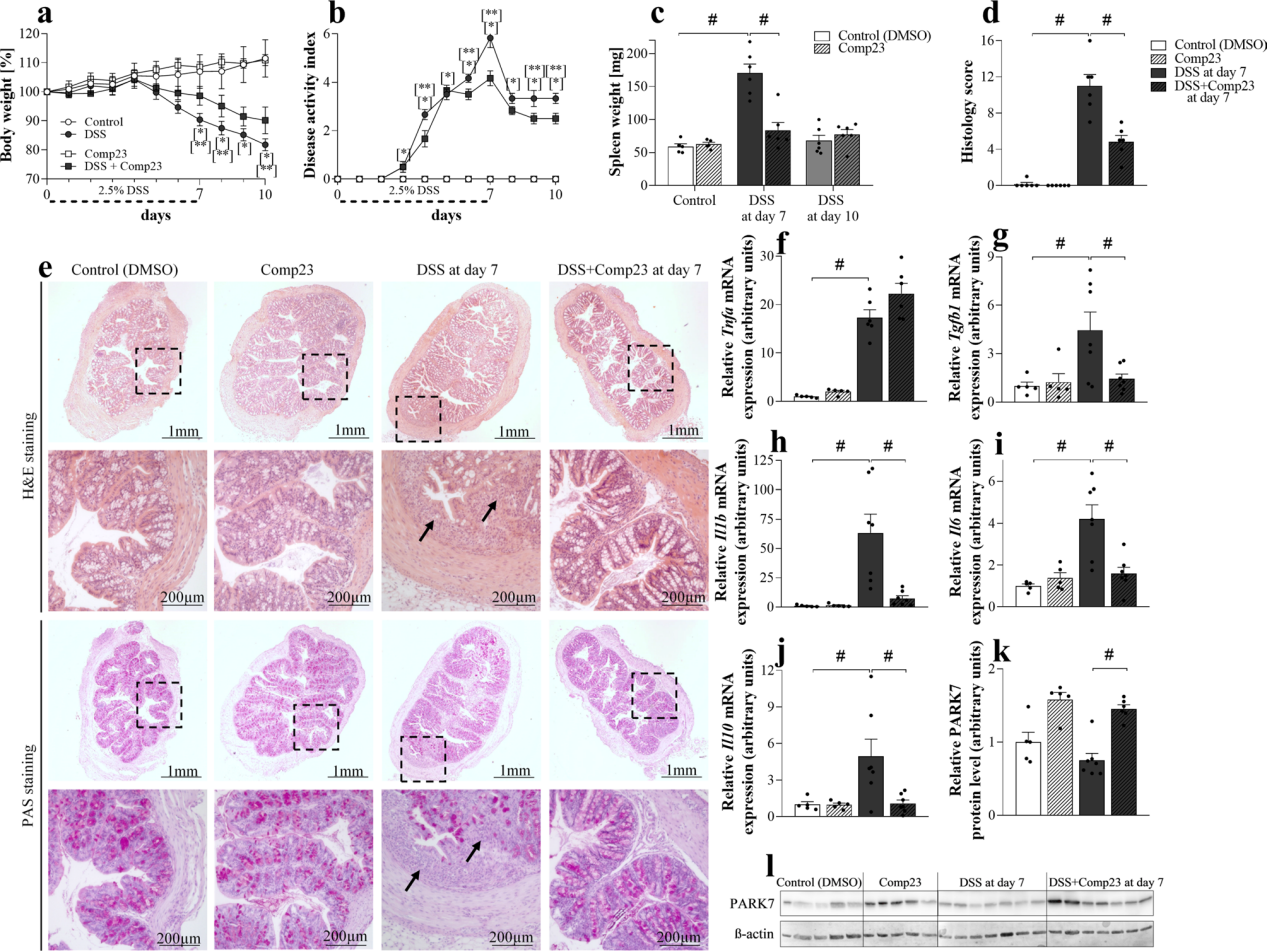
Effect of PARK7-binding Comp23 on DSS induced colitis of mice. In each treatment group body-weight loss (**a**) and disease activity index (**b**) were investigated for 10 days, spleen samples (**c**) were removed and weighed on the 7th and 10th days, HE and PAS staining and histological examination (**d**, **e**), and the molecular biological measurements (**f**–**l**) were done from colon samples removed on the 7th day of the experiment (n = 5–7). Black arrows indicate the accumulated inflammatory cells and damaged crypts and microvilli. Scale bar: 1 mm or 200 µm. The mRNA expression of *Tnfa* (**f**), *Tgfb1* (**g**), *Il1b* (**h**), *Il6* (**i**) and *Il10* (**j**) was determined by real-time PCR (**f**–**j**), and the protein level of PARK7 (**k**, **l**) by Western blot (**k**, **l**) (n = 5–7). Relative expression or amount of PARK7 are presented in comparison with *Rn18S* mRNA (**f**–**j**) or ß-actin protein (**k**, **l**) as internal control and normalized to Control group, respectively. Results are illustrated as mean + SEM, dots represent individual values. Analysis of significance was performed by two-way ANOVA test. *p < 0.05 Control *vs*. DSS at given day, **p < 0.05 DSS *vs*. DSS + Comp23 at given day, ^#^p < 0.05 comparing the connected groups. Full-length blots are included in “Supplementary Informations”.

Intraperitoneal administration of Comp23 reduced body-weight loss (Fig. 5a) and spleen enlargement (Fig. 5c) and improved DAI (Fig. 5b) of DSS compared to vehicle-treated mice. In accordance with the clinical picture Comp23 treatment also reduced histological lesions in the colon of the DSS-treated mice (Fig. 5d,e). Indeed, Comp23 treatment reduced subepithelial immune cell infiltration and mucosal damage, hereby preserved the appearance of crypts and microvilli of DSS-treated mice (Fig. 5e).

Comp23 treatment increased the amount of PARK7 in the colon of DSS-treated mice compared to that of control, Comp23 or DSS-treated mice (Fig. 5k,l).

Comp23 treatment decreased DSS induced colonic expression of *Tgfb1, Il1b, Il6* and *Il10* (Fig. 5g–j), however it had no effect on the expression of *Tnfa* (Fig. 5f).

### Effect of PARK7-binding Comp23 on the intestinal permeability of mice

**Figure 6:**
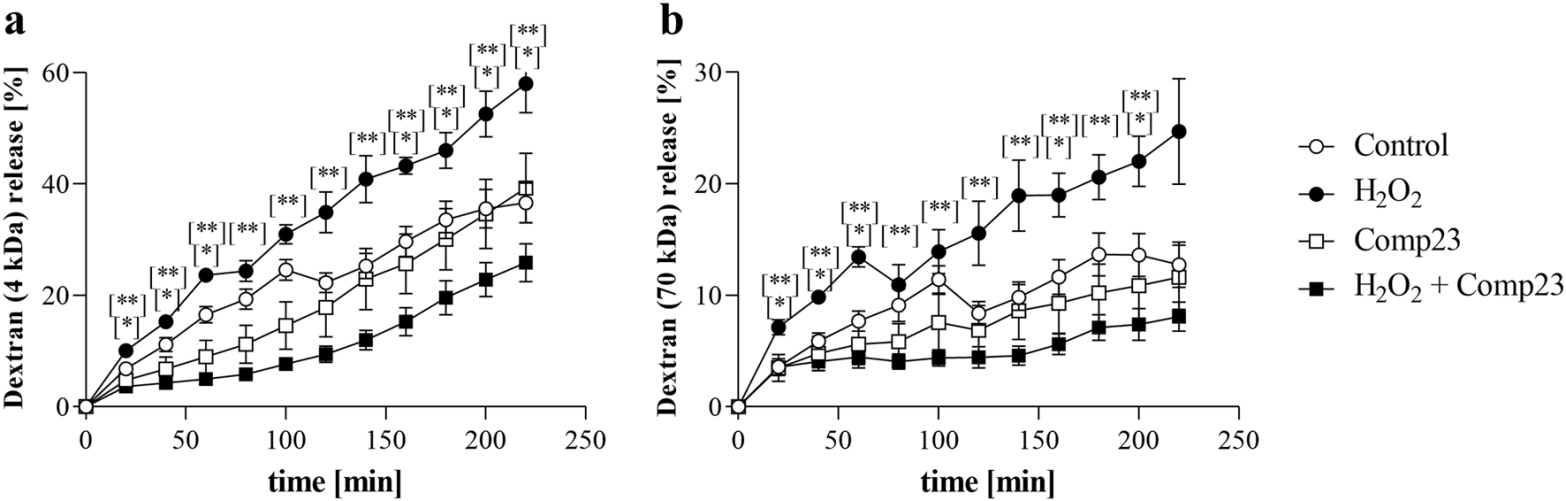
Effect of PARK7-binding Comp23 on the intestinal permeability of WT mice. Mucosal permeability of colon sacs derived from control and Comp23 pretreated WT mice, filled with fluorescence dextrans (4 kDa (**a**) and 70 kDa (**b**)) diluted in DMEM were investigated in the absence or presence of H_2_O_2_ (1000 μM). Permeation of dextrans were measured in every 20 min for 4 h. Results are presented as the percent of total release and illustrated as mean ± SEM. Analysis of significance was performed by two-way ANOVA test. *p < 0.05 Control *vs*. H_2_O_2_; **p < 0.05 H_2_O_2_ *vs*. Comp23 + H_2_O_2_ at given time.

The H_2_O_2_ induced permeability of colon sacs, derived from Comp23 treated mice, to 4 kDa (Fig. 6a) and 70 kDa (Fig. 6b) dextrans was less than that of the sacs derived from vehiculum treated control mice.

## Discussion

Previously, involvement of PARK7 was described in disorders affecting the central nervous system, including Parkinson’s^4^ or Alzheimer’s disease^22^. However, there are a few studies investigating the role of PARK7 in gastrointestinal diseases. Indeed, recently our research group demonstrated the role of PARK7 in the maintenance of duodenal mucosal integrity of children with coeliac disease^12,13^, and Di Narzo^23^ and Zhang et al.^24^ investigated the amount of PARK7 in the plasma and intestine of adult patients with IBD, respectively. PARK7 was also shown to play a role in the pathomechanism of premature piglet model of enteral feeding induced necrotizing enterocolitis^25^, and in ketoprofen-induced oxidative damage of intestinal mucosa^26^.

In the present study, we demonstrated increased amount of PARK7 in the epithelial and lamina propria cells of macroscopically inflamed and non-inflamed mucosa of therapy-naive paediatric patients with CD compared to that of controls (Fig. 1). Interestingly, mucosal PARK7 level of children with UC remained at the level of controls. Our results are differing from that of Di Narzo^23^ who found higher PARK7 level in the plasma of UC compared to CD patients or that of Zhang et al.^24^, demonstrating decreased amount of PARK7 in intestinal samples of patients with IBD. However, these studies are hardly comparable with our one, because of the different type of samples, patients or methods. Indeed, Di Narzo investigated the plasma proteome of adult IBD patients, who were being treated, using a SOMAmer-based high throughput capture array^23^. Less is known about the biopsy specimens and their parameters used by Zhang et al., who investigated the PARK7 immunopositivity of paraffin-embedded colon sections of IBD patients^24^.

Nevertheless, our present study demonstrates significant difference between mucosal PARK7 level of children with CD and UC. Although our observation needs further, large scale confirmation, we hope that determination of mucosal amount of PARK7 may contribute to the easier differential diagnosis of CD and UC in the future, especially in children with. The importance of our finding is particularly highlighted by the fact that the accurate diagnosis of the indeterminate forms of IBD often takes years^27,28^. One can hypothesize, that the observed difference may be due to the well-known immunological differences between the different forms of IBD. Indeed, while CD is characterized by transmural inflammation and the activation of Th1 and Th17 cells^29,30^, UC is characterized by Th2/NKT cell dominance and inflammation affecting the mucosal surface^31,32^.

To explore the underlying mechanisms responsible for the regulation of PARK7 synthesis more deeply, we investigated the effect of different IBD-related factors, including IL-17, TNF-α, TGF-β, H_2_O_2_ or LPS on PARK7 expression by HT-29 colonic epithelial cells (Fig. 2). In these experiments we demonstrated that all investigated factors influenced the synthesis of PARK7. However, we found that while IL-17 or H_2_O_2_ treatment increased, TNF-α, TGF-β or LPS treatment decreased the PARK7 expression by HT-29 cells.

To the best of our knowledge, present study is the first demonstrating the role of IL-17 in the regulation of PARK7 synthesis. IL-17 is the main secreted cytokine of the Th17 cells and plays a central role in the pathomechanism of IBD. Indeed, previous studies demonstrated the increased expression of IL-17 in the inflamed mucosa and sera of patients with IBD^33–35^. Moreover, the expression of mucosal IL-17 correlates with disease activity index, endoscopic and histological scores of active IBD patients^36^. Therefore, further investigating the effect of IL-17 on the PARK7 synthesis we found that while it increased in the colon of DSS treated WT mice, it remained unchanged in the colon of *Il17*−/− mice with DSS induced colitis (Fig. 3c–e). It should be noted that according to the literature, similarly to the observed alteration of mucosal PARK7, the number of Th17 cells and also the expression of IL-17 is elevated in the mucosa of CD patients compared to that of UC patients, suggesting the possible role of IL-17 in the regulation of PARK7 synthesis in vivo^33,35^.

It was shown that IL-17 induces the production of ROS through the activation of NADPH–oxidase^37^ and also that ROS is a potent inducer of PARK7 synthesis^38–40^. In accordance with these literary data, we found that administration of DPI, an inhibitor of NADPH oxidases, inhibits the IL-17 induced synthesis of *PARK7* by HT-29 cells (Fig. 2e), underlying the relevance of ROS in the regulation of PARK7 synthesis.

In our further experiments, we demonstrated that TGF-β treatment resulted in decreased synthesis of PARK7 by HT-29 colon epithelial cells (Fig. 2c,f). Previously, Del Zotto B. et al. found increased TGF-β production in the lamina propria mononuclear cells of UC patients compared to CD patients or controls^41^. Therefore, it is conceivable that increased mucosal production of TGF-β in UC patients may contribute to the decreased presence of PARK7 in their colonic tissue observed in our study.

Finally, environmental factors, including infections are thought to alter the barrier function of intestinal mucosa, thus promoting the development of IBD^42–44^. Bacterial LPS is recognized by innate immune system via Toll-like receptor 4 (TLR4), leading to increased secretion of immunoregulatory molecules, including TNF-α^45,46^. In the present study, we demonstrated that both LPS and TNF-α treatment decreased the PARK7 synthesis of HT-29 cells (Fig. 2c,d,f,g). These results are in accordance with that of Khasnavis et al., who have found that LPS treatment inhibits the expression of PARK7 in primary human neurons and astrocytes^47^.

Taken together all the above mentioned differences regarding the intestinal inflammation of patients with CD or UC may explain at least in part the observed difference in their mucosal amount of PARK7.

In the next set of experiments, we investigated the biological role of PARK7 in mucosal inflammation. First, we examined the effect of PARK7 on the synthesis of IBD-related inflammatory cytokines of intestinal epithelial cells in vitro. We found that gene silencing of *PARK7* significantly alters the expression of many inflammation-related factors (Fig. 4). Indeed, we found that expression of TNF-α increased in *PARK7* gene silenced HT-29 cells (Fig. 4b). Interestingly, the expression of TNF-α was even higher in the PARK7 knockdown HT-29 cells treated with IL-17 compared to IL-17 treated or control HT-29 cells (Fig. 4d,e). Similarly, increased expression of *TNFA* was observed in DSS-treated *IL17*−/− mice (Fig. 3b)—in which the expression of PARK7 remained unchanged (Fig. 3c–e)—compared to WT mice, suggesting that PARK7 plays a determinative role in regulation of TNF-α in vitro and , in vivo as well. There are only few literary data regarding role of PARK7 in the regulation of inflammatory factors. However, similarly to our results Kim et al. described that antigen-induced TNF-α synthesis is higher in mast cells isolated from *Park7*−/− mice as compared to those isolated from WT^48^.

Our data also demonstrated increased *TGFB* expression in PARK7 gene silenced cells, which is in line with the results of Gao et al. who found that PARK7 suppresses TGF-β/Smad pathway, and partially restores pulmonary arterial hypertension of rats^49^.

Moreover, we found that in the absence of PARK7, the expression of other inflammatory cytokines, including *IL1B* and *IL6* was decreased (Fig. 4b). Further investigating the effect of *PARK7* gene silencing, we also examined its effect on *IL1* and *IL6* expression in IL-17 treated HT-29 colon epithelial cells. Indeed, we found that similarly to our previous experiment, *PARK7* gene silencing decreased *IL1B* expression of IL-17 treated, but it had no effect on *IL6* expression (Fig. 4d). One reason of this latter discrepancy can be that while IL-17 treatment had only a little effect on the *IL1B* expression, it increased significantly by about fivefold the expression of *IL6*, which may have suppressed the effect of *PARK7* gene silencing. We must also note that in contrast with our results Chien CH et al. observed elevated IL-1β level in lung of *Park7*−/− mice and in *Park7* knockdown macrophages, as well^50^.

Taken together our data demonstrate, that PARK7 synthesis is regulated by a number of factors playing a central role in the pathomechanism of IBD, and conversely PARK7 itself can influence the synthesis of several IBD related inflammatory factors.

Based on these data it is easy to accept that PARK7 is an important player in the pathomechanism of IBD. Therefore, in the following experiment, we examined the biological effects of the recently developed PARK7 binding Comp23 on DSS-induced mouse model of IBD^19^. The PARK7 specific effect of Comp23 was evidenced in *Park7*−/− cells and mice where the protective, antioxidant effects of the compound were ceased^19,51^. Indeed, Comp23 is thought to prevent the excessive oxidation of 106 cysteine residue of PARK7, thus retaining its activity^51^.

In our in vivo experiments, Comp23 treatment significantly improved clinical symptoms of DSS induced colitis, it reduced body-weight loss and improved DAI values, as well (Fig. 5a,b). Our results are in line with observation of Zhang et al. who observed worse clinical symptoms in DSS-treated *Park7*−/− than in DSS treated WT mice^24^. Moreover, in accordance with the clinical symptoms, Comp23 treatment reduced enlargement of spleen, preserved normal appearance of crypts and microvilli and decreased the number of immune cells in colonic subepithelial layer of DSS-treated mice (Fig. 5c–e). To further investigate the significance of PARK7 in the mucosal pathology, we examined the effect of Comp23 on the H_2_O_2_-treatment induced permeability of ex vivo colon preparations (Fig. 6)*.* In line with our previous experiments on small intestinal sacs^13^, we found that Comp23 greatly decreased the permeability of H_2_O_2_-treated colon sacs (Fig. 6), confirming the determinative role of PARK7 in the maintenance of mucosal integrity.

Investigating the molecular biological processes underlying the better clinical symptoms, we found that Comp23 treatment significantly increased the amount of PARK7 in the colon of DSS-treated mice (Fig. 5k,l). Perhaps it is not so surprising, since previously it has been demonstrated that Comp23 prevents the excessive oxidation of 106 cysteine residue of PARK7, which thus in line with the study of Takahashi-Niki K et al. may inhibit the oxidation induced chaperone-mediated degradation of PARK7^51^.

We also found that Comp23 treatment resulted in decreased expression of *Il1b, Il6* and *Tgfb1* in colonic mucosa of DSS-treated mice (Fig. 5g–i). These cytokines are determinatives in the maturation of Th1^52^, Th17 and Treg^53^ cells, which are responsible for the mucosal inflammation in CD^54^.

However, interestingly Comp23 treatment also decreased expression of *Il10* a potent anti-inflammatory cytokine (Fig. 5j). Considering that in our experiments *Park7* gene silencing did not alter expression of *Il10* (Fig. 4b), we suggest that decreased colonic expression of *Il10* may be rather an indirect consequence of decreased inflammation, observed in Comp23 treated mice with DSS induced colitis.

Finally, we must also note that our results of Comp23 treated mice with DSS-induced colitis are not completely consistent with our in vitro experiments. Although, the observed difference in the expression of *IL1* and *IL6* may be simply due to differences in the complexity of an in vitro and in vivo model, we believe that the main point is different. Indeed, while in our in vitro experiments gene silencing decreased the amount of PARK7 in case of our in vivo experiment Comp23 treatment increased its amount. Moreover, Comp23 treatment by protecting PARK7 from its oxidation may also increase its activity.

In summary, although there are limitations of our study, including the number of the human samples, which do not let us to correlate the mucosal amount of PARK7 with disease activity index or other clinical parameters, we made great progress in the understanding of the biological effects of PARK7 in the pathomechanism of IBD. Indeed, we showed that PARK7 is differentially expressed in the mucosa of children with CD or UC, suggesting that quantifying the expression of PARK7 may help to differentiate between the two main clinical forms of IBD. Moreover, our in vitro experiments demonstrated that IBD related factors influence the synthesis of PARK7, suggesting that their local balance may be responsible for the observed differences in mucosal PARK7 level of children with CD and UC, as well. We also showed that presence of PARK7 alters the expression of many IBD-related factors in vitro. Finally, in the present study we demonstrated for the first time that PARK7 binding Comp23 treatment reduces mucosal inflammation and clinical symptoms of DSS-induced colitis, suggesting its possible therapeutic value. Taken together, our present work contributes to better understanding the role of PARK7 in mucosal inflammation and may facilitate the development of new therapeutics to hinder IBD.

## Supplementary Information

Supplementary Information.

## Data Availability

The data used to support the findings of this study are included within the article.
