# Supplementary material for: Immunomodulatory role of Parkinson’s disease 7 in inflammatory bowel disease
Source: Sci Rep. 2021 Jul 16;11:14582. doi: 10.1038/s41598-021-93671-1 (PMC8285373; doi:10.1038/s41598-021-93671-1)
Supplement: Supplementary file 1 — Supplementary Information. [file 41598_2021_93671_MOESM1_ESM.pdf]

Immunomodulatory role of Parkinson's disease 7 in inflammatory bowel disease

Rita Lippai et. al

SUPPLEMENT

Original images of western blot membranes, explanations, sample identifications are detailed below.

After blotting, the nitrocellulose membranes were cut in two pieces based on the molecular weight markers. The investigated proteins (PARK7, ß-actin or GAPDH) from the identical samples were present on separate membrane pieces, avoiding the interference derived from the multiple chemiluminescent signals of different proteins.

Samples included in representative images in the manuscript are highlighted with bold characters and denoted by red boxes.

Figure 1/c

sample order in Figure 1/c:

Control 1, Control 2, Control 3, Control 4, Control 5, Control 6, Control 7 | iCD 1, iCD 2, iCD 3, iCD 4, iCD 5, iCD 6, iCD 7 | niCD 7, niCD 5, niCD 4, niCD 3, niCD 2, niCD 1, niCD 10, niCD 9, niCD 8 | UC 1, UC 2, UC 3, UC 4, UC 5, UC 6, UC 7

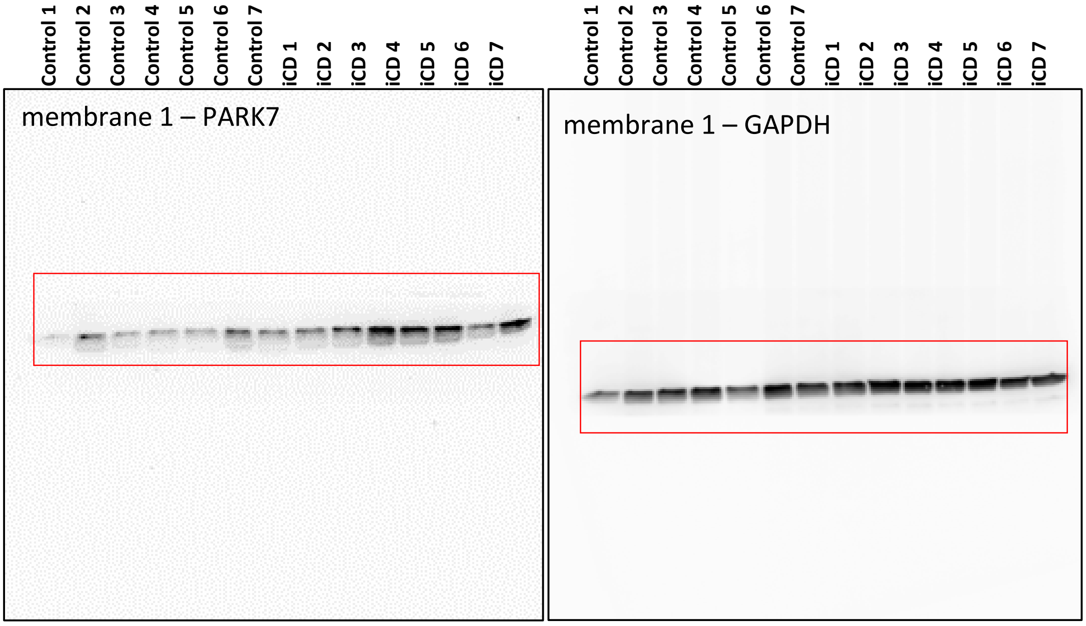

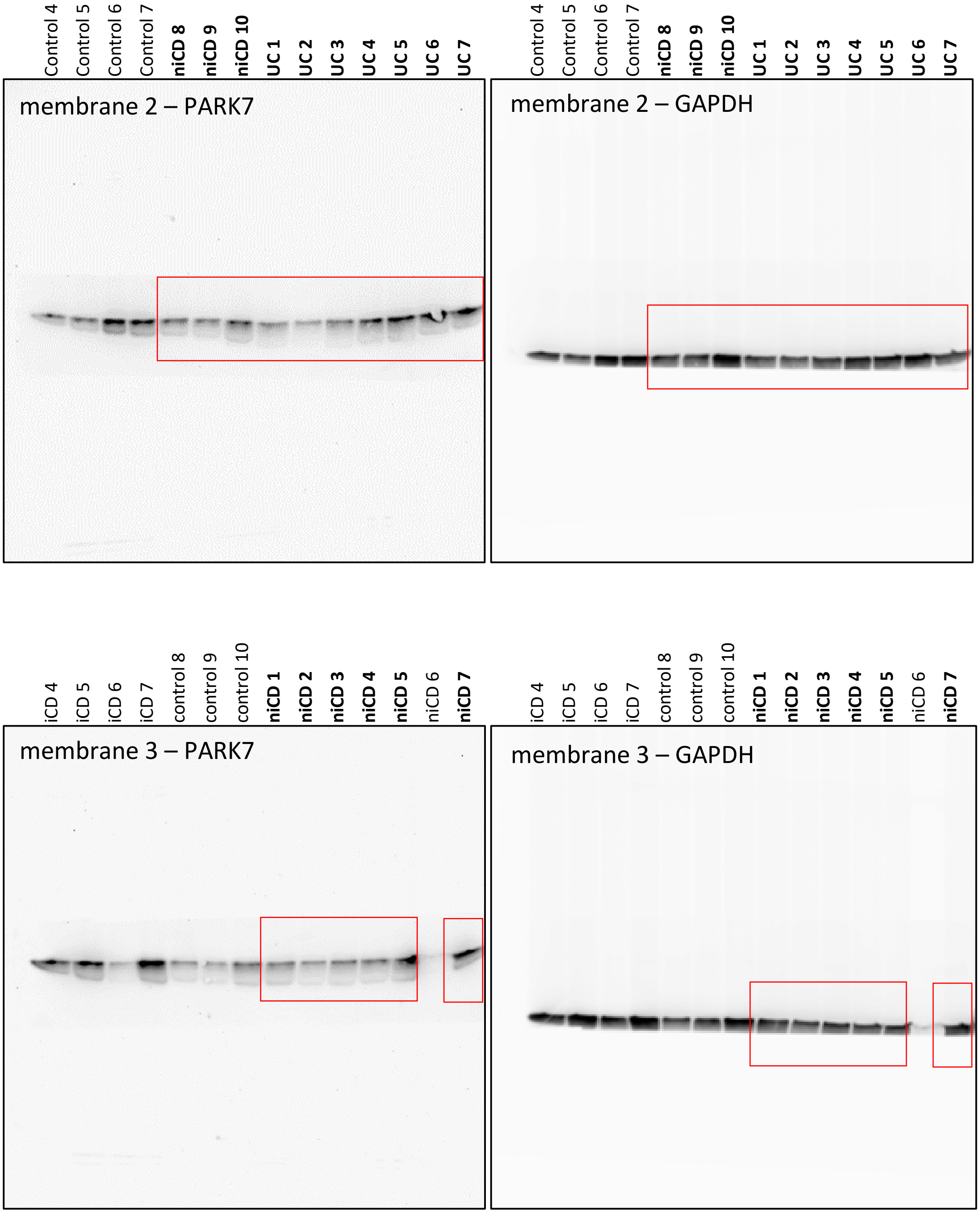
Control 4
Control 5
Control 6
Control 7
niCD 8
niCD 9
niCD 10
UC 1
UC 2
UC 3
UC 4
UC 5
UC 6
UC 7
membrane 2 – PARK7
Control 4
Control 5
Control 6
Control 7
niCD 8
niCD 9
niCD 10
UC 1
UC 2
UC 3
UC 4
UC 5
UC 6
UC 7
membrane 2 – GAPDH
iCD 4
iCD 5
iCD 6
iCD 7
control 8
control 9
control 10
niCD 1
niCD 2
niCD 3
niCD 4
niCD 5
niCD 6
niCD 7
membrane 3 – PARK7
iCD 4
iCD 5
iCD 6
iCD 7
control 8
control 9
control 10
niCD 1
niCD 2
niCD 3
niCD 4
niCD 5
niCD 6
niCD 7
membrane 3 – GAPDH

<u>Figure 2/g</u>

sample order in Figure 2/g:

Control_1, Control_2, Control_3, Control_4, Control_5, Control_6 |

LPS_1, LPS_2, LPS_3, LPS_4, LPS_5, LPS_6

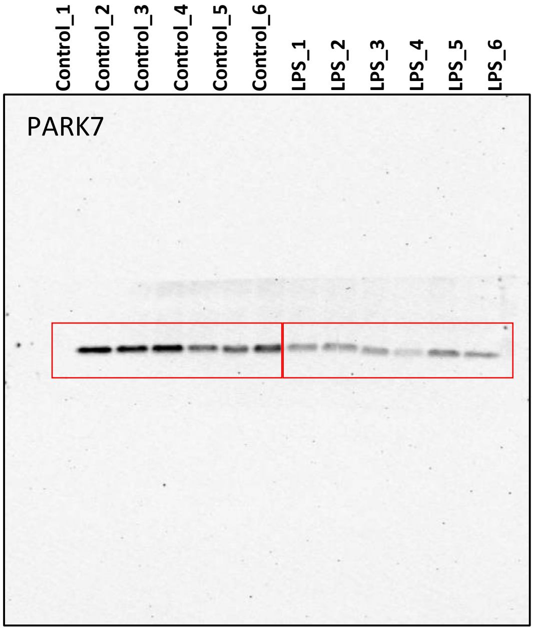

Figure 3/e

sample order in Figure 3/e:

WT → WT0_2, WT0_4, WT0_3, WT0_1, WT0_5 | WT3_3, WT3_4, WT3_5, WT3_6, WT3_1 | WT5_3, WT5_4, WT5_5, WT5_6, WT5_1 | WT7_3, WT7_4, WT7_5, WT7_6, WT7_2

IL17 -/- → KO0_2, KO0_3, KO0_4, KO0_5, KO0_6 | KO3_2, KO3_4, KO3_5, KO3_6, KO3_1 | KO5_3, KO5_4, KO5_5, KO5_6, KO5_1 | KO0_7, KO7_3, KO7_4, KO7_5, KO7_6, KO7_2

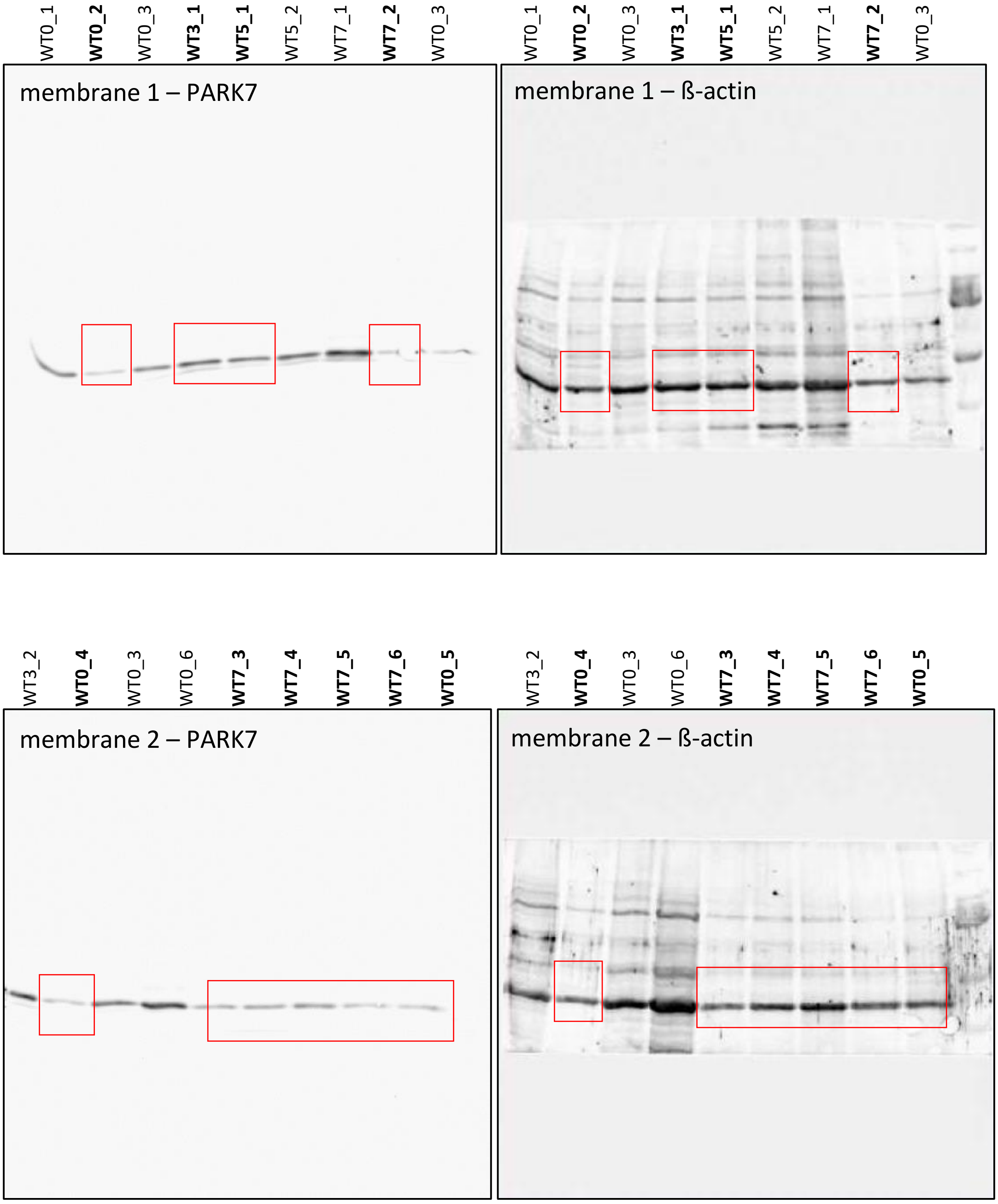

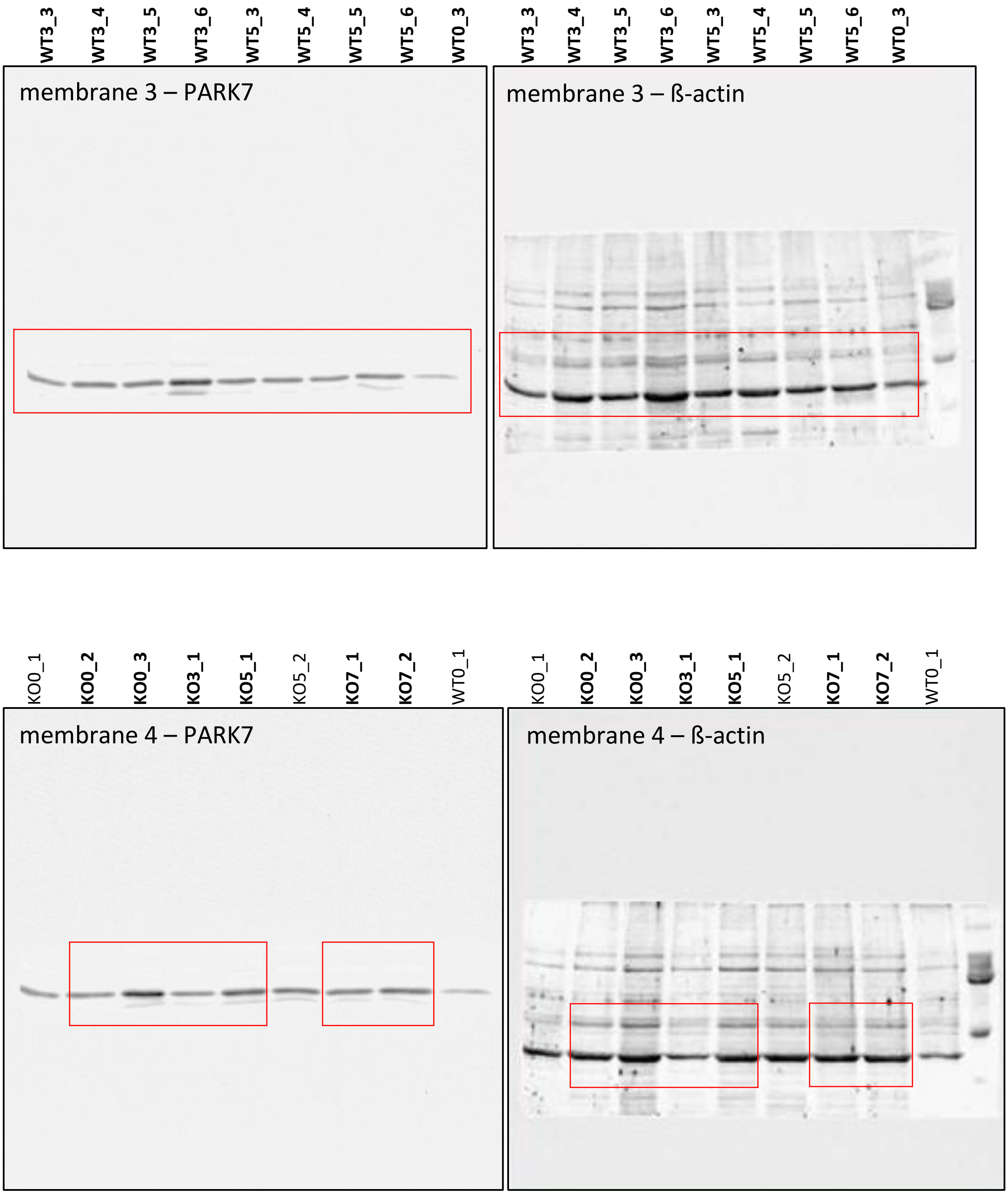
WT3_3
WT3_4
WT3_5
WT3_6
WT5_3
WT5_4
WT5_5
WT5_6
WT0_3
membrane 3 – PARK7
WT3_3
WT3_4
WT3_5
WT3_6
WT5_3
WT5_4
WT5_5
WT5_6
WT0_3
membrane 3 – ß-actin
KO0_1
KO0_2
KO0_3
KO3_1
KO5_1
KO5_2
KO7_1
KO7_2
WT0_1
membrane 4 – PARK7
KO0_1
KO0_2
KO0_3
KO3_1
KO5_1
KO5_2
KO7_1
KO7_2
WT0_1
membrane 4 – ß-actin

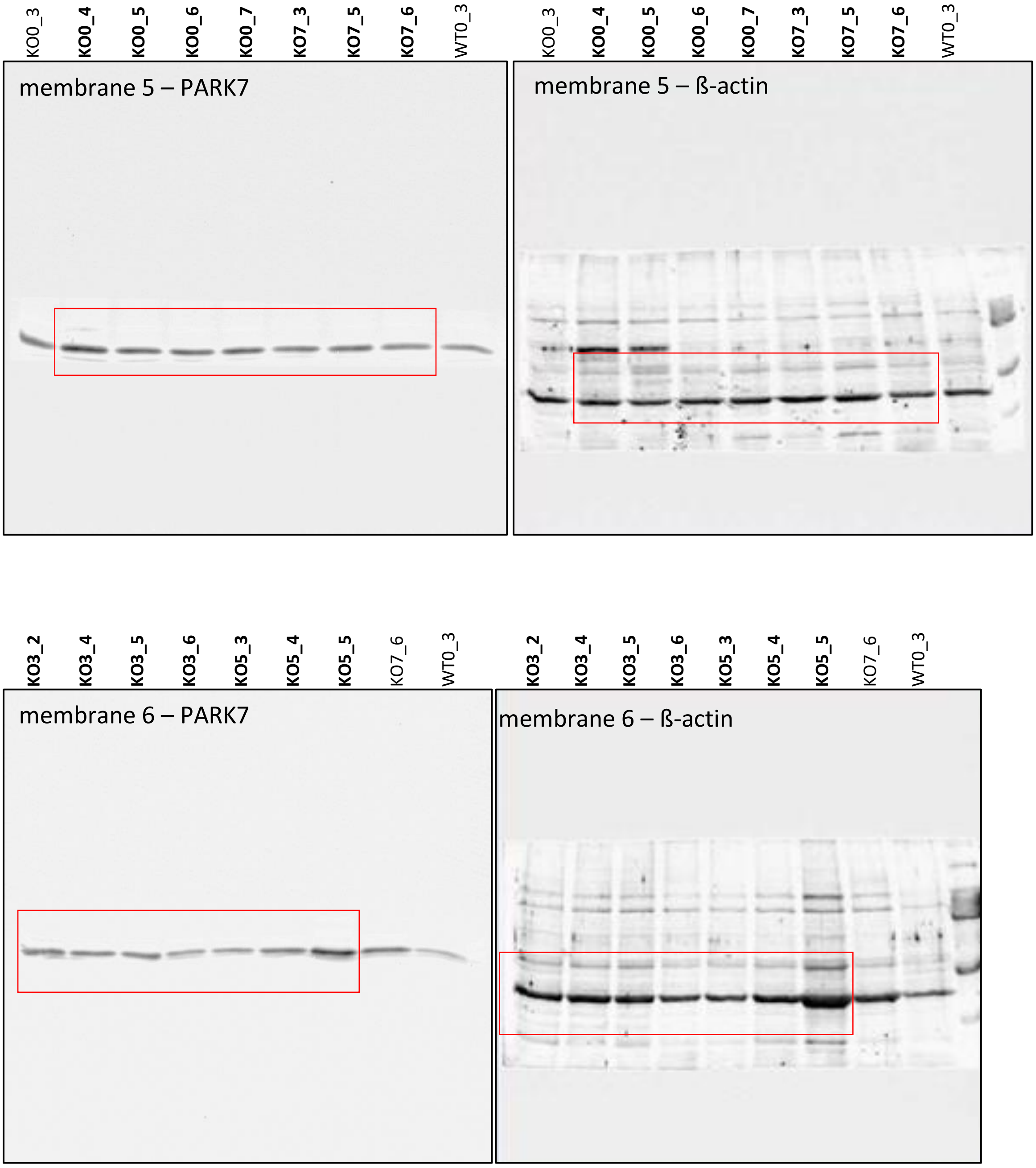
KO0_3
KO0_4
KO0_5
KO0_6
KO0_7
KO7_3
KO7_5
KO7_6
WT0_3
membrane 5 – PARK7
KO0_3
KO0_4
KO0_5
KO0_6
KO0_7
KO7_3
KO7_5
KO7_6
WT0_3
membrane 5 – ß-actin
KO3_2
KO3_4
KO3_5
KO3_6
KO5_3
KO5_4
KO5_5
KO7_6
WT0_3
membrane 6 – PARK7
KO3_2
KO3_4
KO3_5
KO3_6
KO5_3
KO5_4
KO5_5
KO7_6
WT0_3
membrane 6 – ß-actin

Figure 4/f

sample order in 4/f:

scRNA_1, scRNA_2, scRNA_3, scRNA_4, scRNA_5 | siRNA_1, siRNA_2, siRNA_3, siRNA_4, siRNA_5

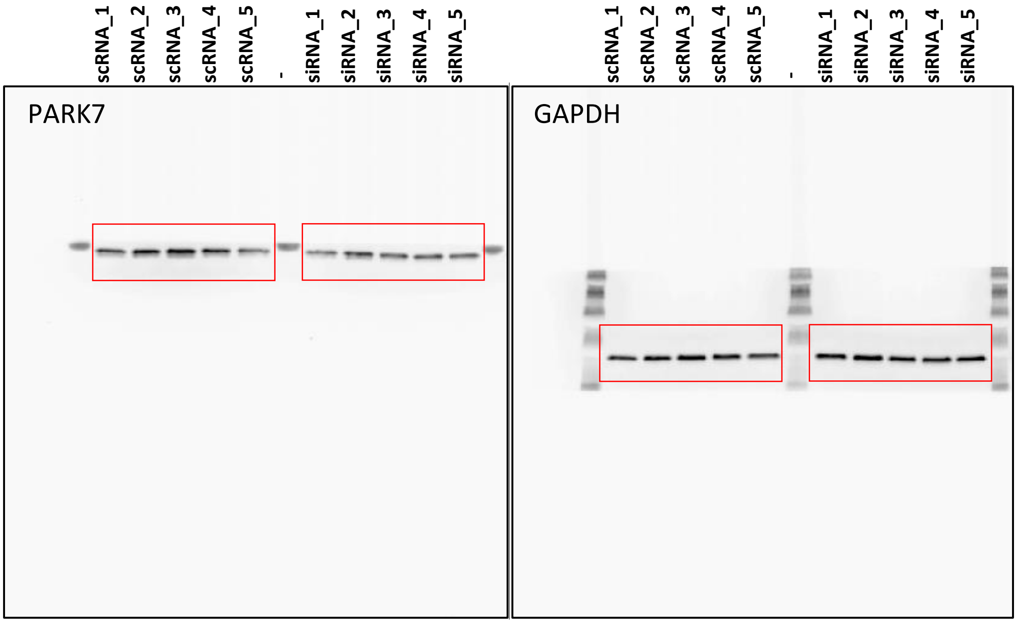

Figure 4/g

sample order in 4/g:

scRNA_1, scRNA_2, scRNA_3, scRNA_4 | scRNA+IL17_1, scRNA+IL17_2, scRNA+IL17_3, scRNA+IL17_4, scRNA+IL17_5 | siRNA+IL17_1, siRNA+IL17_2, siRNA+IL17_3, siRNA+IL17_4, siRNA+IL17_5

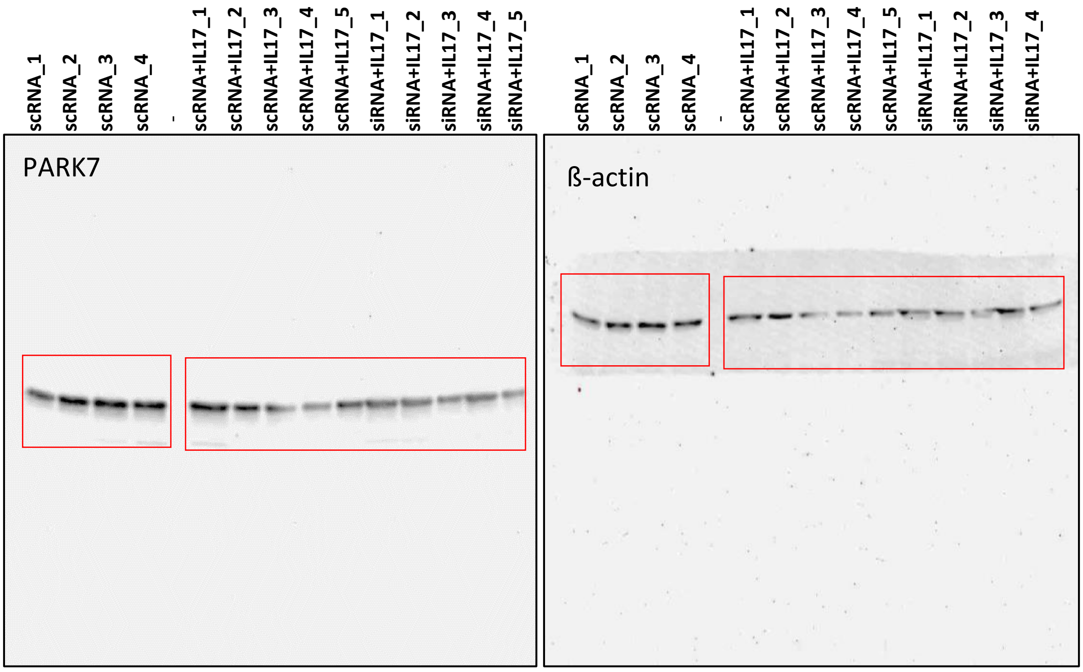

Figure 5/I

sample order in Figure 5/I:

Control (DMSO)_1, Control (DMSO)_2, Control (DMSO)_3, Control (DMSO)_4, Control (DMSO)_5 | Comp23_1, Comp23_2, Comp23_3, Comp23_4, Comp23_5 | DSS at day 7_1, DSS at day 7_2, DSS at day 7_3, DSS at day 7_4, DSS at day 7_5, DSS at day 7_6, DSS at day 7_7 | DSS+Comp23 at day 7_1, DSS+Comp23 at day 7_2, DSS+Comp23 at day 7_3, DSS+Comp23 at day 7_4, DSS+Comp23 at day 7_5, DSS+Comp23 at day 7_6, DSS+Comp23 at day 7_7

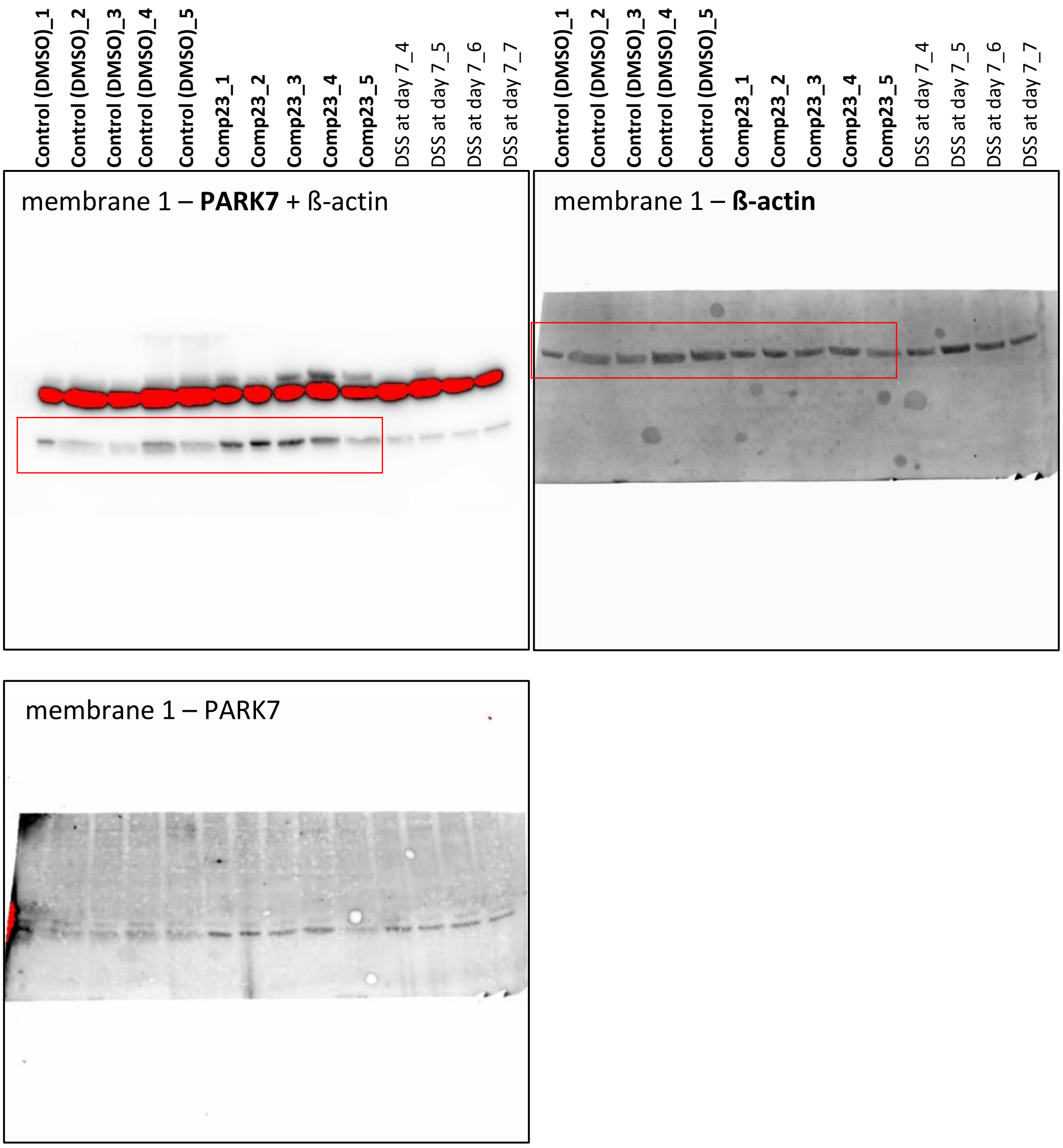

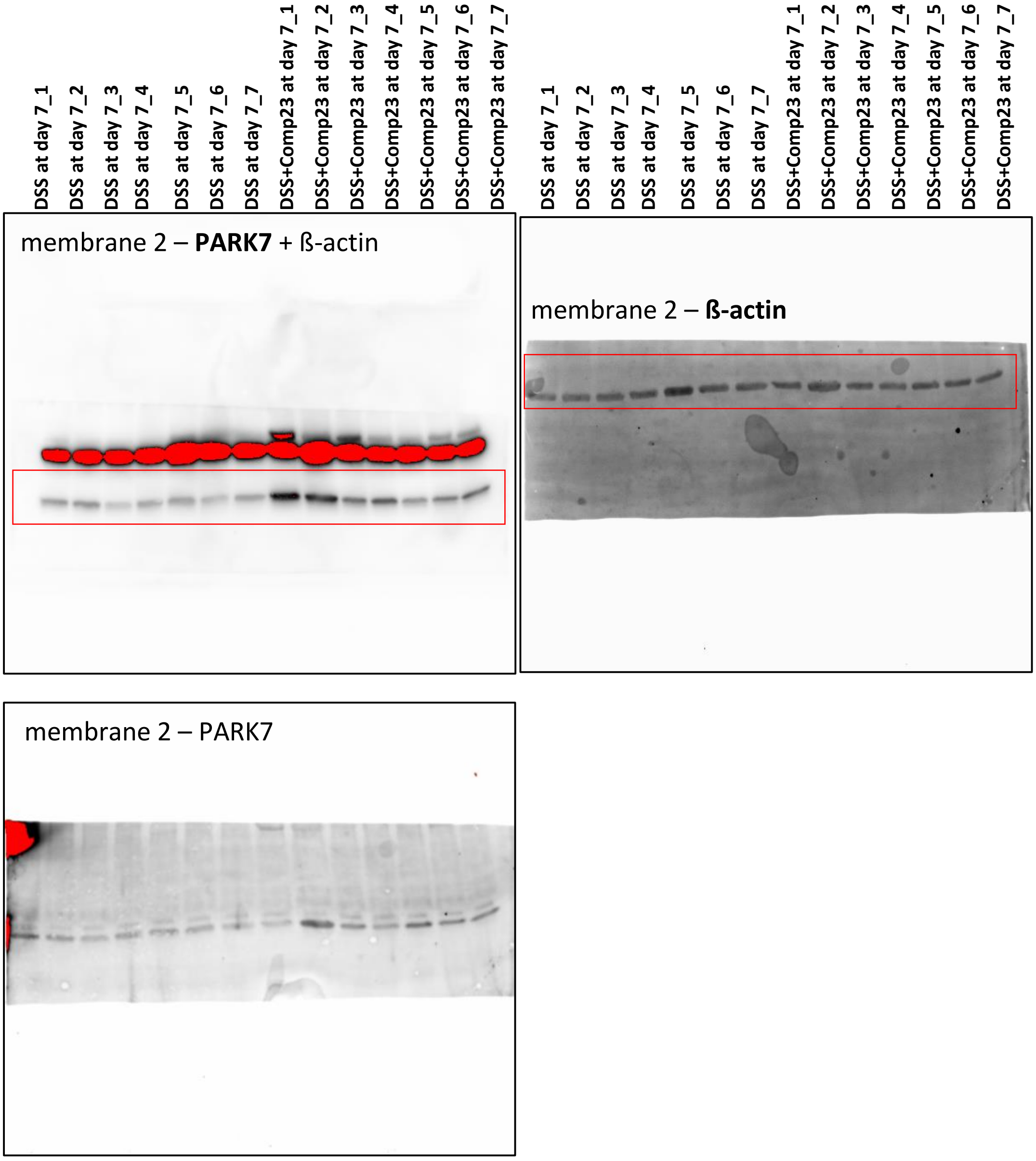
DSS at day 7_1
DSS at day 7_2
DSS at day 7_3
DSS at day 7_4
DSS at day 7_5
DSS at day 7_6
DSS at day 7_7
DSS+Comp23 at day 7_1
DSS+Comp23 at day 7_2
DSS+Comp23 at day 7_3
DSS+Comp23 at day 7_4
DSS+Comp23 at day 7_5
DSS+Comp23 at day 7_6
DSS+Comp23 at day 7_7
membrane 2 – PARK7 + ß-actin
DSS at day 7_1
DSS at day 7_2
DSS at day 7_3
DSS at day 7_4
DSS at day 7_5
DSS at day 7_6
DSS at day 7_7
DSS+Comp23 at day 7_1
DSS+Comp23 at day 7_2
DSS+Comp23 at day 7_3
DSS+Comp23 at day 7_4
DSS+Comp23 at day 7_5
DSS+Comp23 at day 7_6
DSS+Comp23 at day 7_7
membrane 2 – ß-actin
membrane 2 – PARK7
